# Distribution of nerve fibers and nerve-immune cell association in mouse spleen revealed by immunofluorescent staining

**DOI:** 10.1038/s41598-020-66619-0

**Published:** 2020-06-17

**Authors:** Dailun Hu, Huda A. M. Al-Shalan, Zhongli Shi, Penghao Wang, Yongkang Wu, Philip K. Nicholls, Wayne K. Greene, Bin Ma

**Affiliations:** 1 Clinical College, Hebei Medical University, Shijiazhuang, 050020 China; 2 Medical, Molecular and Forensic Sciences, Murdoch University, Murdoch, 6149 Australia; 3 Department of Microbiology/Virology, College of Veterinary Medicine, Baghdad University, Baghdad, 10071 Iraq; 4 Department of Laboratory Medicine, West China Hospital, Sichuan University, Chengdu, 610041 China

**Keywords:** Haemic and immune systems, Nervous system

## Abstract

The central nervous system regulates the immune system through the secretion of hormones from the pituitary gland and other endocrine organs, while the peripheral nervous system (PNS) communicates with the immune system through local nerve-immune cell interactions, including sympathetic/parasympathetic (efferent) and sensory (afferent) innervation to lymphoid tissue/organs. However, the precise mechanisms of this bi-directional crosstalk of the PNS and immune system remain mysterious. To study this kind of bi-directional crosstalk, we performed immunofluorescent staining of neurofilament and confocal microscopy to reveal the distribution of nerve fibers and nerve-immune cell associations inside mouse spleen. Our study demonstrates (i) extensive nerve fibers in all splenic compartments including the splenic nodules, periarteriolar lymphoid sheath, marginal zones, trabeculae, and red pulp; (ii) close associations of nerve fibers with blood vessels (including central arteries, marginal sinuses, penicillar arterioles, and splenic sinuses); (iii) close associations of nerve fibers with various subsets of dendritic cells, macrophages (Mac1^+^ and F4/80^+^), and lymphocytes (B cells, T helper cells, and cytotoxic T cells). Our data concerning the extensive splenic innervation and nerve-immune cell communication will enrich our knowledge of the mechanisms through which the PNS affects the cellular- and humoral-mediated immune responses in healthy and infectious/non-infectious states.

## Introduction

The human nervous system includes the central nervous system (CNS; containing the brain and spinal cord) and the peripheral nervous system (PNS; containing the sensory (somatic sensory and visceral sensory) and motor (somatic motor divisions and the autonomic nervous system (ANS; including sympathetic, parasympathetic, and enteric components)))^1^. Recent studies have demonstrated that bi-directional communication/interaction between the nervous system and the immune system plays a crucial role in the host’s responses to pathogen invasion, tissue injury, and other homeostatic dangers/threats^1–5^. The functional organization of the neural control of the immune system is based on principles of reflex regulation^6^. For example, the immune system is regulated by the CNS through the secretion of hormones from the pituitary and other neuroendocrine/endocrine organs^7,8^ and by the PNS through local nerve (fiber)-immune cell interactions. The PNS can regulate the development, deployment, and homeostatic regulation of the immune system^3^. This type of local neuroimmune interaction involves the “hardwiring” of sympathetic/parasympathetic (efferent) and visceral sensory (afferent) nerves to primary and secondary lymphoid tissue/organs^4–6^. Neurotransmitters (e.g., acetylcholine, norepinephrine, and histamine) and neuropeptides (e.g., vasoactive intestinal peptide and substance P) released from nerve terminals (or even from the immune cells) might modulate the immune response in homeostasis and diseases^9^. Cytokines and other immunologic factors synthesized and released by the immune cells (e.g., macrophages, dendritic cells (DCs), and lymphocytes) or even by neurons/glial cells can also have significant effects on the activities/functions of the PNS^9^.

The spleen is separated into two main compartments, namely the blood-containing red pulp (primarily for innate immunity) and the lymphoid cells-containing white pulp (primarily for adaptive immunity) by an interface called the marginal zone^10^. In the red pulp, macrophages efficiently remove pathogens, dead cells/cellular debris, and aging erythrocytes. In the white pulp, a highly-organized lymphoid structure is responsible for the local and systemic regulation of immunity^10^. Accumulating evidence suggests that an intricate communication exists between the PNS and the spleen, and that this crosstalk might play an essential role in the regulation of the immune response^9,11–17^. Therefore, an understanding of the splenic innervation by both autonomic (efferent) and sensory (afferent) fibers is crucial for a better appreciation of the response of the spleen to immune challenge and tissue injury.

Sympathetic norepinephrine fibers enter the spleen via the splenic nerve, and much of the network is closely associated with the splenic artery and its branches into the spleen^18–20^. These sympathetic nerve fibers might have close associations with lymphocytes, macrophages, and DCs^14–16^. In addition, sensory innervation of the spleen has also been reported^11,13^. However, the suggestion of parasympathetic innervation and/or control of the spleen has continued to be controversial, ever since acetylcholine was first isolated from the spleen^11,13,20,21^.

The information on the innervation and nerve-immune interactions within the spleen remains very limited, given the studies described above. We have demonstrated in our previous study the existence of non-myelinating Schwann cells and Remak fibers (including small nociceptive (C-type) axons, postganglionic sympathetic axons, and some preganglionic sympathetic/parasympathetic fibers) inside the mouse spleen^22^. In the present study, a rabbit anti-neurofilament heavy (NF-H) antibody was utilized as a reliable marker to characterize the nerves/nerve fibers inside the mouse spleen. Neurofilaments (NFs) are intermediate filaments particularly abundant in axons, where they are essential for the radial growth of axons during development, the maintenance of axon caliber, and the transmission of electrical impulses along axons^23^. NFs are composed of four subunits (neurofilament light (NF-L), neurofilament middle (NF-M), NF-H, and α-internexin (or peripherin)), each having different domain structures and functions. In addition, NFs might play a role in intracellular transport to axons and dendrites^23^. In the adult nervous system, NFs in small unmyelinated axons contain more peripherin and less NF-H, whereas NFs in large myelinated axons contain more NF-H and less peripherin.

NF-H has been utilized as a cellular marker for the characterization of nerve/nerve fibers in the lymphoid organs (e.g., thymus, lymph node, and spleen) across several species (e.g., human and rat)^19,22,24,25^. By using immunofluorescent staining and confocal microscopy/three-dimensional (3D) reconstruction, we have now investigated the distribution of nerve fibers and PNS-immune cell relationship *in situ* in the mouse spleen to improve our knowledge of the microanatomical basis of bi-directional communication of the PNS and secondary lymphoid tissue/organs (e.g., spleen, lymph nodes, and gut-associated lymphoid tissue).

## Results

### Distribution of nerve fibers in the mouse spleen

A rabbit anti-NF-H antibody was used as a reliable marker to label the nerve fibers inside the spleen. This antibody only recognized a protein of 220 KD, which is the mass of NF-H^26^. To validate this antibody, we also performed immunofluorescent staining on a few types of mouse tissues (e.g., brain, skin, liver, and small intestine) and observed brightly stained cells/fibers with clear morphology that is expected for the nerves/nerve fibers in these tissues (Supplementary Fig. 1). For negative control experiments, no staining was observed when only three secondary antibodies were applied (Supplementary Fig. 2).

**Figure 1:**
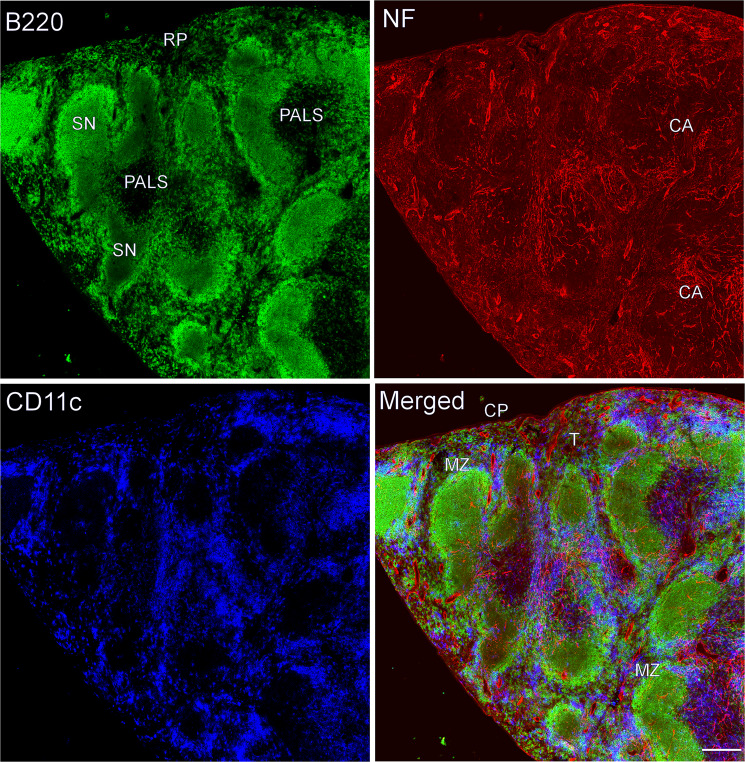
Overview of splenic innervation of a C57BL/6 mouse. Antibodies against NF-H (red), B220 (green), and CD11c (blue) detect mainly nerve fibers, B cells, and DCs, respectively. CA: central artery; CP: capsule; SN: splenic nodule; RP: red pulp; T: trabecula; MZ: marginal zone; PALS: periarteriolar lymphoid sheath; Objective lens: 40×; Scanning mode: Tile scan; Scale bar: 200 µm.

**Figure 2:**
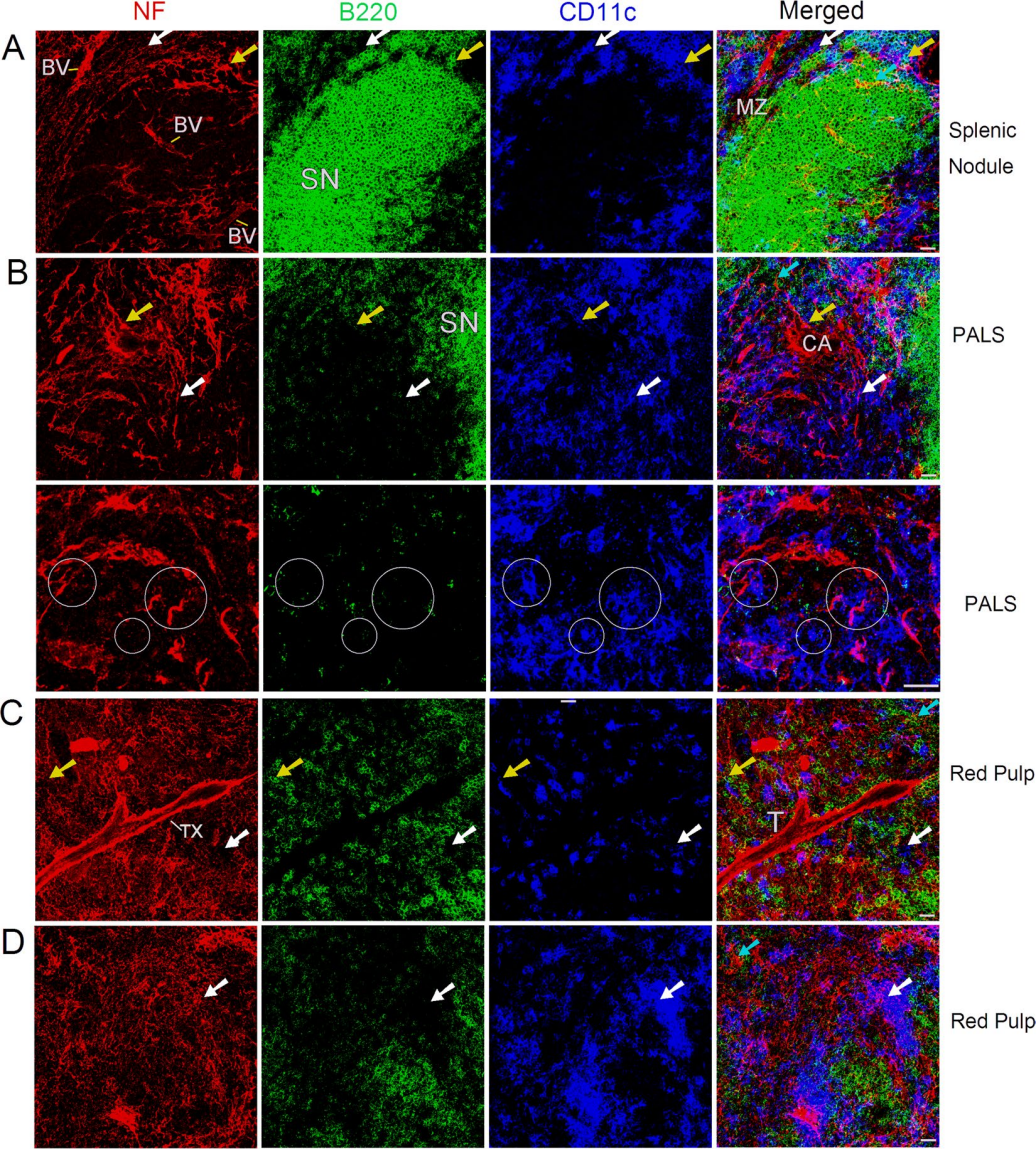
Distribution of nerve fibers, B cells, and DCs in splenic nodule/marginal zone (**A**), PALS (**B**), and red pulp (**C,D**) of a C57BL/6 mouse spleen. Antibodies against NF-H (red), B220 (green), and CD11c (blue) detect mainly nerve fibers, B cells, and DCs, respectively. The cyan arrows indicate B220^+^ B cells closely associated with nerve fibers. B220^-^CD11c^+^ DCs closely apposed to nerve fibers were shown by white arrows. The yellow arrows indicate B220^+^CD11c^+^ DCs closely associated with nerve fibers. (**B**) Images in the second row (high-resolution views of the image cropped from the first row) show close associations (indicated by white circles) with nerve endings (appearing as red dots) and immune cells in PALS. (**C**) Trabecular plexus travels along the trabecula. Each micrograph is a maximal intensity projection of a Z-Stack. Stack size: 6.0 µm; optical slice interval: 0.50 µm. BV: blood vessel; MZ: marginal zone; SN: splenic nodule; CA: central artery; PALS: periarteriolar lymphoid sheath; T: trabecula; TX: trabecular plexus; Objective lens: 40×; Scale bar: 20 µm.

We found an extensive meshwork of nerve fibers in splenic compartments including the capsule, splenic nodules (B cell follicles), marginal zones, periarteriolar lymphoid sheath (PALS), and red pulps (Figs. 1 and 2). The intensity of nerve fibers varied in the various parts of the spleen. For example, if sectioned transversely, the middle portion of spleen had more innervation than other portions of the spleen (e.g., tips of the spleen, data not shown).

The splenic nodules (Fig. 2A) had fewer nerve fibers compared with the PALS (Fig. 2B) and red pulp (Fig. 2C). The marginal zone (Fig. 2A) contained extensive nerve fibers that were closely associated with marginal B cells and DCs. In the PALS (Fig. 2B), an extensive network of nerve fibers ran along the central artery, formed plexi around it, and extended into the PALS and splenic nodules. We also observed that many nerve fibers had close associations with B220^+^ B cells and with B220^-^CD11c^+^/B220^+^CD11c^+^ DCs.

In the spleen two types of nerve-immune cell associations have been observed. The nerve fiber-immune cell association was regarded as the first, and nerve ending (appearing as small red dots)-immune cell association was the second (Fig. 2B). These two types of associations were also observed in the splenic red pulp (Fig. 2C,D).

### Relationship of nerve fibers and immune cells in the mouse spleen

**Figure 3:**
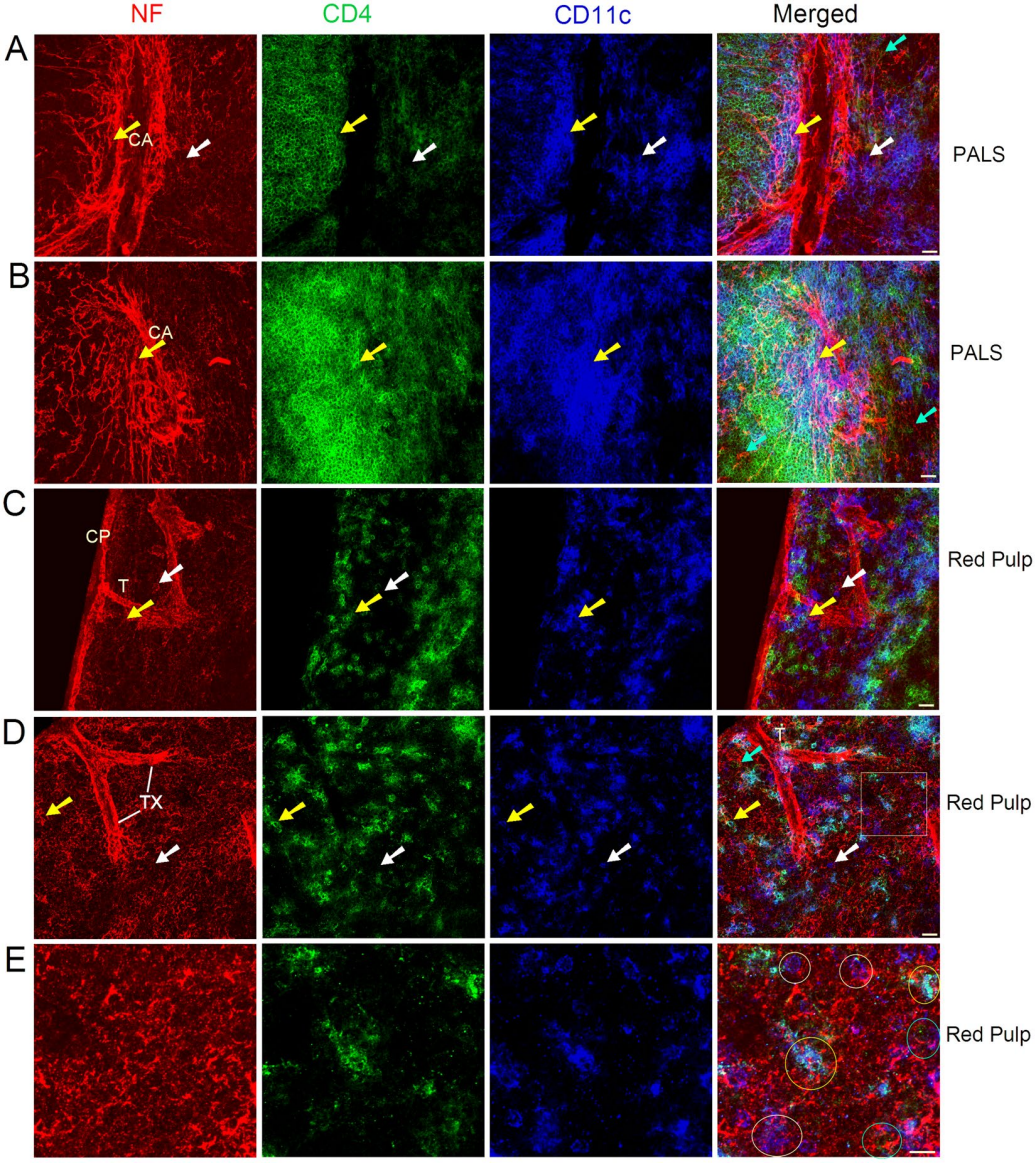
Distribution of nerve fibers, T helper cells, and DCs in the PALS (**A,B**), and red pulp (**C–E**) of a C57BL/6 mouse spleen. Antibodies against NF-H, CD4, and CD11c detect mainly nerve fibers (red), T helper cells (green), and DCs (blue), respectively. CD4^+^ T helper cells that are closely associated with nerve fibers are shown with cyan arrows. CD4^−^CD11c^+^ DCs closely apposed to nerve fibers are shown with white arrows. CD4^+^ CD11c^+^ DCs having a close association with nerve fibers are indicated by yellow arrows. (**A,B**) Nerve fibers form a nerve plexus around the central arteries and extend into the PALS. (**E**) High-resolution view of a cropped region (shown as a square) from (**D**) show close associations with nerve endings (appear as red dots) with CD4^+^ T helper cells (cyan circles), CD4^−^CD11c^+^ DCs (white circles), and CD4^+^CD11c^+^ DCs (yellow circles). Each micrograph is a maximal intensity projection of a Z-Stack. Optical slice interval: 0.50 µm; Stack size: 6.0 µm; CA: central artery; PALS: periarteriolar lymphoid sheath; CP: capsule; T: trabecula; TX: trabecular plexus; Objective lens: 40×; Scale bar: 20 µm.

We then investigated the distribution of nerve fibers, T helper cells (by anti-CD4 staining), and DCs inside the mouse spleen; the results are shown in Fig. 3 and Supplementary Fig. 3 (for an overview). Only some T helper cells were seen in the splenic nodules (Supplementary Fig. 3). In the PALS, an extensive network of nerve fibers (plexus) was observed around the central arteries, and these nerve fibers exhibited close associations with many CD4^+^ T helper cells and CD4^−^CD11c^+^/CD4^+^CD11c^+^ DCs (Fig. 3A,B). In addition, in the spleen red pulp, the nerve fibers exhibited close associations with CD4^+^ T helper cells and CD4^−^CD11c^+^/CD4^+^CD11c^+^ DCs (Fig. 3C–E).

**Figure 4:**
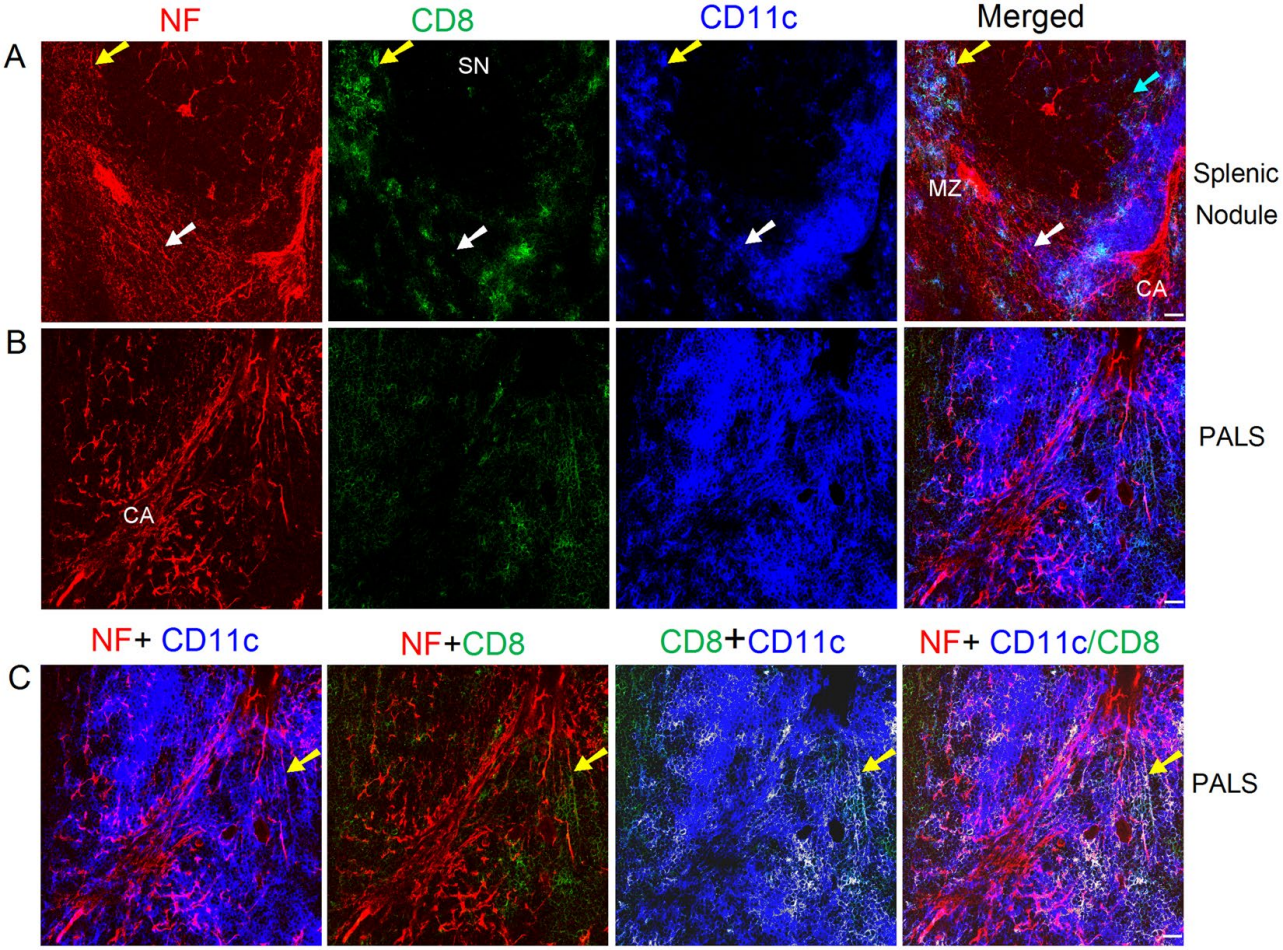
Distribution of nerve fibers, cytotoxic T cells, and DCs in splenic nodule/marginal zone (**A**) and PALS (**B,C**) of a C57BL/6 mouse spleen. Antibodies against NF-H (red), CD8a (green), and CD11c (blue) detect mainly nerve fibers, cytotoxic T cells, and DCs, respectively. The cyan arrows indicate CD8a^+^ cytotoxic T cells that are closely associated with nerve fibers. The white arrows show a CD8a^−^CD11c^+^ DC closely apposed to nerve fibers. The yellow arrows indicate CD8a^+^ CD11c^+^ DCs having a close association with nerve fibers. (**A**) A few sparse nerve fibers together with many nerve endings (red dots) are present in the splenic nodule. Each micrograph is a maximal intensity projection of a Z-Stack. Stack size: 6.0 µm; optical slice interval: 0.50 µm. (**C**) CD8a^+^ CD11c^+^ DCs appear white after colocalization analysis in the third and fourth panels. CA: central artery; PALS: periarteriolar lymphoid sheath; MZ: marginal zone; SN: splenic nodule; Objective lens: 40×; Scale bar: 20 µm.

We also checked the distribution of nerve fibers, cytotoxic T cells (by anti-CD8a immunofluorescence), and DCs in the spleen. Only very few cytotoxic T cells were observed in splenic nodules (Fig. 4A). Extensive nerve fibers occurred in the splenic marginal zones, and some of these fibers exhibited close associations with marginal zone cytotoxic T cells and CD8a^−^CD11c^+^/CD8a^+^CD11c^+^ DCs. In addition, the extensive nerve fibers around the central artery also exhibited close appositions to CD8a^+^ cytotoxic T cells and CD8a^−^CD11c^+^/CD8a^+^CD11c^+^ DCs (Fig. 4B,C) in the PALS.

**Figure 5:**
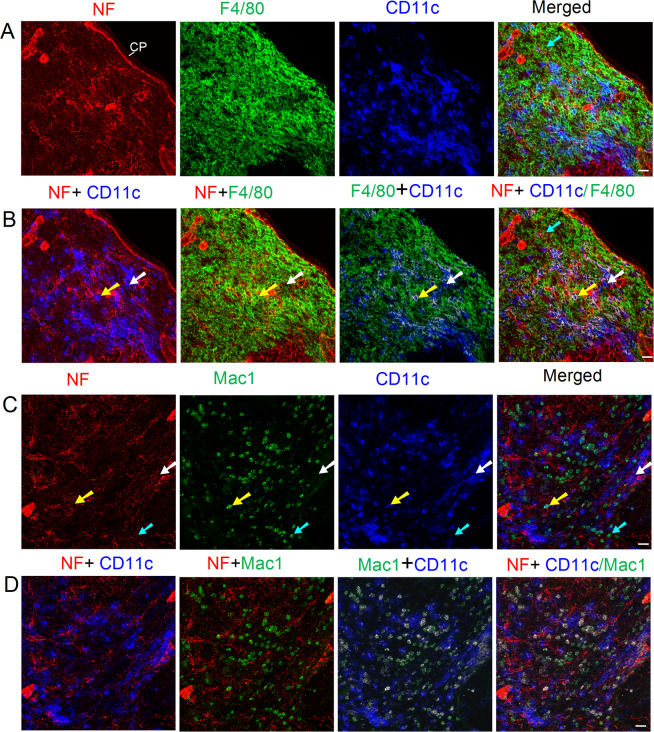
Distribution of nerve fibers, macrophages, and DCs in the red pulp of a C57BL/6 mouse spleen. Antibodies against NF-H (red), F4/80 or Mac1 (green), and CD11c (blue) label mainly nerve fibers, red pulp macrophages, and DCs, respectively. (**A,B**) The cyan arrows indicate an F4/80^+^CD11c^−^ macrophage that is closely associated with nerve fibers. The white arrows show an F4/80^−^CD11c^+^ DC closely apposed to nerve fibers. The yellow arrows indicate an F4/80^+^CD11c^+^ DC having a close association with nerve fibers. (**B**) F4/80^+^CD11c^+^ DCs are labeled with white color pixels after colocalization analysis. (**C**) The cyan arrows indicate a Mac1^+^CD11c^low^ macrophage that is closely associated with nerve fibers. The white arrows show a Mac1^−^CD11c^+^ DC closely apposed to nerve fibers. The yellow arrows indicate Mac1^+^CD11c^+^ DC having a close association with nerve fibers. (**D**) Mac1^+^CD11c^+^ DCs are marked in white after colocalization analysis. Each micrograph is a maximal intensity projection of a Z-Stack. Optical slice interval: 0.50 µm; Stack size: 6.0 µm; CP: capsule; SP: subcapsular plexus; Objective lens: 40×; Scale bar: 20 µm.

Two-way communication of macrophages and nerves can trigger, maintain, and terminate the macrophage reactions^3^. Therefore, the association of nerves and two subsets of splenic red pulp macrophage, namely F4/80^+^ macrophages and Mac1^+^macrophages, was investigated; the results are shown in Fig. 5. The two subsets of macrophages were located in the splenic red pulp only. An extensive network of nerve fibers was seen in the red pulp (including the trabecula), and these nerve fibers were closely associated with F4/80^+^CD11c^−^ macrophages, F4/80^−^CD11c^+^/F4/80^+^CD11c^+^ DCs, Mac1^+^ CD11c^low^ macrophages, and Mac1^−^CD11c^+^ DC/ Mac1^+^CD11c^+^ DCs (Fig. 5).

### Associations of nerve fibers with blood vessels in the mouse spleen

**Figure 6:**
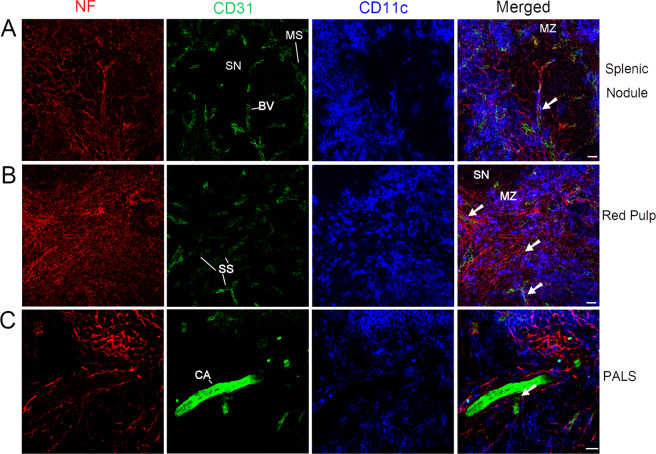
Distribution of nerve fibers, blood vessels, and DCs in the splenic nodule/marginal zone (**A**), red pulp (**B**), and PALS (**C**) of a C57BL/6 mouse spleen. Antibodies against NF-H (red), CD31 (green), and CD11c (blue) detect mainly nerve fibers, blood vessels, and DCs, respectively. (**A,B**) Each micrograph is a maximal intensity projection of a Z-Stack. Optical slice interval: 0.50 µm; Stack size: 6.0 µm; The white arrows show the close associations with nerve fibers and blood vessels. SN: splenic nodule; MS: marginal sinus; BV blood vessel; MZ: marginal zone; SS: splenic sinus; CA: central artery; PALS: periarteriolar lymphoid sheath; Objective lens: 40×; Scale bar: 20 µm.

Splenic blood circulation is open since afferent arterial blood ends in sinusoids surrounding the white pulp^10^. Blood flows into venous sinuses through sinusoidal spaces and red pulp, collecting into efferent splenic veins. To comprehend the connection between the nerve fibers and blood vessels/DCs, triple-immunolabelling with anti-CD31 (a blood vessel endothelial cell marker), anti-CD11c, and anti-NF-H antibodies was performed; the results are shown in Fig. 6. In splenic nodules and marginal zones, some blood vessels including the capillaries (containing pericytes) were closely associated with nerve fibers (Fig. 6A). Similar close associations of nerve fibers and blood vessels (including the splenic sinus) were also observed in the red pulp (Fig. 6B) indicating a neuronal control of blood flow inside the splenic red pulp. In addition, a close association of nerve fibers with blood vessels (including central arteries) was also found in the PALS (Fig. 6C).

## Discussion

Branches from the celiac plexus, left celiac ganglion, and the right vagus nerve form the nerve plexi inside the spleen. At the hilum, nerve fibers from the splenic nerve enter the spleen around the splenic artery, travel with the vasculature in the plexi, continue into the spleen along the trabeculae with the trabecular plexi, and extend into the white pulp including the splenic nodules and PALS^18,22^.

In the present study, in the mouse spleen, we have observed an extensive meshwork of nerve fibers, which has not been reported before. Some previous reports have shown that sympathetic (norepinephrine) innervation is particularly rich in T cell zones and in areas of DCs/macrophages, whereas the follicular/nodular zones are poorly innervated^13,18,19,24^. In addition, in these reports, only scattered/sparse nerve fibers primarily associated with plexi along the trabeculae have been observed in the red pulp^13^. However, our study has demonstrated: (a) the presence of nerve fibers in each compartment of the spleen, including the splenic nodules, marginal zones, and red pulp, although the number of nerve fibers in the splenic nodules is much fewer compared with that of other compartments; (b) the intensity of nerve fibers in PALS and marginal zones is much higher than that of nerve fibers in some previous reports^11,18–20^; (c) the intensity of nerve fibers is similar to that of Remak fibers (indirectly demonstrated by non-myelinated Schwann cells) inside the spleen as shown in one of our previous studies^22^.

Since the NF-H is a non-specific neuronal/axonal marker, we should mention that we might see more nerve fibers (e.g., parasympathetic and visceral sensory fibers), except for the tyrosine hydroxylase positive sympathetic nerve fibers, inside the spleen. Additional cellular markers such as tyrosine hydroxylase (for sympathetic nerve fibers), choline acetyltransferase (for cholinergic fibers), calcitonin gene-related peptide (CGRP; for sensory fibers from dorsal root ganglion and motor fibers from anterior horn of spinal cord), and transient receptor potential cation channel subfamily V member 1 (TRPV1) and acetylcholinesterase can be used to identify specific nerve types inside the spleen.

Smooth muscles and pericytes are target cells of peripheral nerves and under the control of PNS. We have also shown that afferent nerve fibers are distributed through/along the trabeculae and closely associated with blood vessels with/without smooth muscles (e.g., the central arteries and their branches, penicillin arterioles, marginal sinuses, and splenic sinuses). The nerve fibers travel along blood vessels, form plexi around the blood vessels, and extend to the parenchyma of spleen. Therefore, the neuronal regulation of blood flow and vascular permeability of blood vessels might affect the subsequent dynamics of immune cells and the clearance of the blood-borne antigens/foreign materials.

Splenic mature B cells have two main populations: follicular B cells (major B cell population) and marginal zone B cells (lining the marginal sinus and bordering the red pulp)^27^. The follicular B cells are mainly responsible for T-cell-dependent immune reactions and marginal B cells can catch blood-borne antigens via complement receptors and stimulate both T-cell-independent and -dependent immune responses^27^.

Some studies have revealed that sympathetic norepinephrine nerve fibers are associated with B/T lymphocytes within splenic white pulp^18,20^. However, these kinds of nerve-B/T cell associations have not been observed in several other studies^24^. We have seen intensive nerve fiber-B cell associations in the splenic nodules (including the germinal centers), PALS, marginal zones, and red pulp. These kinds of close associations show that B cell differentiation/maturation (inside the splenic nodules) and antigen presentation (in the marginal zones) may be regulated by the splenic innervation. We also observed close associations of nerve fibers with CD4^+^ T helper cells and CD8^+^ cytotoxic T cells, indicating a potential regulation of T cell response by the splenic innervation.

Mononuclear phagocytes (e.g., monocytes, macrophages, and DCs) protect the host by identifying pathogens/foreign bodies, removing dead cells/ cell debris, and regulating tissue homeostasis and innate/adaptive immunity^10,28^. Close associations/interactions of nerve fibers and DCs have been observed inside the spleen in some reports^24^. In our previous study, we have also detected close association of a few subsets of DCs with Remak fibers in the splenic white pulp and red pulp^22^. In the present study, we have revealed intensive nerve-DC associations. A few interesting points should be mentioned concerning this type of nerve-DC associations. First, around the central arteries in the PALS, we have found clusters of DCs, many of which have close associations with nerve fibers. Second, nerve fibers are closely apposed to many DCs in PALS and marginal zones. Third, some nerve fibers are also closely apposed to DCs in the red pulp. Fourth, the phenotypes of DCs^28^ associated with nerve fibers include B220^-^CD11c^+^, B220^+^CD11c^+^ (plasmacytoid DCs),CD4^−^CD11c^+^ (lymphoid DCs), CD4^+^CD11c^+^ (lymphoid DCs), Mac1^low^CD11c^+^, Mac1^+^CD11c^+^, F4/80^−^CD11c^+^, and F4/80^+^CD11c^+^ DCs in the spleen. Although further functional *in vitro* and *in vivo* studies need to be carried out, our findings provide a reliable microanatomical basis for the neural regulation/control of the functions of DCs including antigen presentation and cytokine production.

Macrophages in the spleen have two main protective activities during infections of blood-borne pathogens or foreign materials. The first type of well-characterized macrophage is responsible for phagocytosis and the elimination of pathogens from the circulation^10^. The second type of macrophage is defined by the expression of CD markers (e.g., F4/80, Mac1 (CD11b), CD68), and these macrophages play a crucial role in the activation of the immune system^10^. Recent studies have demonstrated local associations of macrophages and nerve fibers in the spleen^18,22^. In our study, we have observed that some nerve fibers have close appositions with Mac1^+^CD11c^low^ and F4/80^+^CD11c^−^ macrophages in the red pulp, suggesting that splenic nerves might regulate the macrophage functions (e.g., antigen presentation and cytokine production).

Nerves and nerve fibers are comparatively static structures associated with blood vessels, while most immune cells are wandering cells inside tissues or organs. Therefore, this type of nerve-immune cell contacts or association, which may be defined as neuro-immune cell unit or neuro-immune synapse, should be dynamic^29,30^. This neuro-immune cell unit or synapse has some features of neurological and immune synapses^29,30^. Two types of cells can communicate either through direct ligand-receptor binding or through neurotransmitters or/and inflammatory mediators. Further functional studies are helpful in revealing the detailed mechanisms of this type of cell-to-cell communication.

Firstly, high-resolution confocal imaging/3D reconstruction, electron microscopy, and immune electron microscopy can be applied to confirm the presence of the “synapse” or synapse-like association if some critical requirements for a classical synapse are met^29,30^. In our previous study, we have analysed neuro-immune cell membrane-membrane contact by using high-resolution confocal imaging and quantitative colocalization analysis^31^. Some other studies utilizing electron microscopy have observed neuroimmune synapse with a synaptic cleft about 6nm^32^. Secondly, the effects of neurotransmitters or neuropeptides on immune cells should be studied. In our previous study, we have seen the expression of muscarinic acetylcholine receptor (subtype M2) on lymphocytes and DCs in mouse *Peyers’* patches^33^. Some other studies also showed that neurotransmitters such as acetylcholine and dopamine regulated the functions of B/T cells and DC/macrophages^34,35^. Some neurotransmitters are immunosuppressive, while others may stimulate and activate immunity^35,36^. Thirdly, inflammatory mediators such as pro-inflammatory cytokines, prostaglandins, serotonin, and histamine from immune cells (even from neural cells or glial cells) can have variable effects on nerves of PNS^37,38^.

In summary, our novel findings concerning extensive splenic innervation and its relationship with immune cells should shed some light on the microanatomical basis of the bi-directional crosstalk of the PNS and secondary lymphoid tissue/organs in health and diseases. Certainly, further *in vivo* and *in vitro* molecular and functional studies need to be carried out to undercover the mystery of this bi-directional communication^39^. Furthermore, chemical, pharmacological, electrical, or other manipulations of these neuroimmune interactions should benefit the development of potential practical therapeutic approaches for certain neurological, neuroimmunological, infectious, and immunological diseases^39–43^.

## Methods

### Animals and sectioning

Eight male C57BL/6 mice (age from 8–10 weeks) were bought from the Animal Resources Centre (Murdoch, Australia). All experiments were carried out in accordance with Australian national rules and approved by the Murdoch University Animal Ethics Committee (permit number: R2700/14). 20-µm-thick cryosections of mouse spleen, brain, skin, liver, and small intestine were produced and mounted on poly-L-lysine-coated microscope slides (Sigma, Castle Hill, Australia) as described^22^.

### Antibodies

The detailed information about antibodies is listed in Table 1.

**Table 1:** Specificities and sources of primary and secondary antibodies.

Target [Cata. no.]	Conjugate	Species and isotype	Main cells labeled	Dilution	Company
CD11c [60002]	—	Armenian Hamster monoclonal IgG	Dendritic cell	1:500	STEMCELL Technologies (Tullamarine, Australia)
B220 (CD45R) [103202]	—	Rat monoclonal IgG	B cell	1:300	Australian Biosearch (Karrinyup, Australia)
CD31 [102402]	—	Rat monoclonal IgG	Blood vessel endothelial cells	1:300	Australian Biosearch
CD4 [100506]	—	Rat monoclonal IgG	CD4^+^ Thymocytes	1:300	Australian Biosearch
CD8a [100802]	—	Rat monoclonal IgG	CD8a^+^ Thymocytes	1:300	Australian Biosearch
Mac1(CD11b) [100302]	—	Rat monoclonal IgG	Macrophages	1:300	Australian Biosearch
F4/80 [123102]	—	Rat monoclonal IgG	Macrophages	1:300	Australian Biosearch
Neurofilament 200 (NF-H) [N4142]	—	Rabbit polyclonal	Neuronal marker	1:2000	Sigma (St. Louis, Missouri, US)
Rabbit IgG H&L [ab150078]	Alexa Fluor 555	Goat polyclonal	—	1:1000	Abcam Australia (Melbourne, Australia)
Armenian Hamster IgG H&L [ab173004]	Alexa Fluor 647	Goat polyclonal	—	1:1000	Abcam Australia
Rat IgG H&L [ab150157]	Alexa Fluor 488	Goat polyclonal	—	1:1000	Abcam Australia

### Immunofluorescence labeling of sections

Following a brief wash with phosphate-buffered saline (PBS), we fixed the cryosections with 4% paraformaldehyde (PFA, Electron Microscopy Sciences, Hatfield, PA, USA) for 15 min at room temperature. After a brief wash with PBS, the sections were treated with 0.2% Triton X-100 (Sigma) in PBS for 5 min. To block potential non-specific binding sites on the tissues, we incubated the sections with 2% goat serum (Sigma) in PBS for 30 min. Primary antibodies (diluted in 2% goat serum) were then used to treat the slides (overnight at 4 °C). After 3 times of washing (5 min each), slides were then treated with secondary antibodies (diluted in 2% goat serum) for 2 h at room temperature. Following 3 times of washing (5 min each), we mounted the section in Fluorescence Mounting Medium (Dako, Sydney, Australia) with glass coverslips.

### Confocal imaging

Slides were analysed by a Nikon C2 Plus Confocal Microscope (Nikon Instruments, Melville, NY, USA). Three lasers (488 nm, 561 nm, and 633 nm) were used to excite the fluorescent dyes, and a Plan Apo λ 40× objective lens was utilized for all the imaging. The operating program of confocal system was NIS-Elements AR program. We obtained pictures from a large area of section by using the Tile Scan mode. Optic sectioning was performed, and 3D reconstruction was done by using the Maximal Intensity Projection of a Z-Stack. After the 3D projection, compared with 2D images, the images were brighter, which, however, did not mean overexposure or saturation. The brightness and contrast of the pictures were then adjusted by using NIS-Elements AR program. We then utilized the Corel PaintShop Pro 2018 (Corel Corporation, Ottawa, Canada) to crop and label our images to generate the final version of the pictures.

### Analysis of colocalization

We used ImageJ 1.51w for the analysis of colocalization. After two 24-bit pictures (copied from the green and blue channels) were transformed into two 8-bit images (grayscale), the “Colocalization” command was run to generate the results. The white pixels in the final resulted pictures represented the colocalized pixels.

## Supplementary information

Supplementary Information.

## Data Availability

All data generated or analysed during this study are included in this published article and its Supplementary Information files.

## References

[1] Kenney MJ, Ganta CK (2014). Autonomic nervous system and immune system interactions. Compr. Physiol..

[2] Tian L, Ma L, Kaarela T, Li Z (2012). Neuroimmune crosstalk in the central nervous system and its significance for neurological diseases. J. Neuroinflamm..

[3] Ordovas-Montanes J (2015). The regulation of immunological processes by peripheral neurons in homeostasis and disease. Trends Immunol..

[4] Gabanyi I (2016). Neuro-immune interactions drive tissue programming in intestinal macrophages. Cell.

[5] Yoo BB, Mazmanian SK (2017). The enteric network: interactions between the immune and nervous systems of the gut. Immunity.

[6] Pavlov VA, Chavan SS, Tracey KJ (2018). Molecular and functional neuroscience in immunity. Annu. Rev. Immunol..

[7] ThyagaRajan S, Priyanka HP (2012). Bidirectional communication between the neuroendocrine system and the immune system: relevance to health and diseases. Ann. Neurosci..

[8] Soto-Tinoco E, Guerrero-Vargas NN, Buijs RM (2016). Interaction between the hypothalamus and the immune system. Exp. Physiol..

[9] Pacheco, R., Contreras, F. & Prado, C. Cells, molecules and mechanisms involved in the neuro-immune interaction in Cell Interaction (ed. Gowder, S.) 139–166 (InTech, 2012).

[10] Bronte V, Pittet MJ (2013). The spleen in local and systemic regulation of immunity. Immunity.

[11] Nance DM, Sanders VM (2017). Autonomic innervation and regulation of the immune system (1987-2007). Brain Behav. Immun..

[12] Gautron L (2013). Neuronal and nonneuronal cholinergic structures in the mouse gastrointestinal tract and spleen. J. Comp. Neurol..

[13] Jung WC, Levesque JP, Ruitenberg MJ (2017). It takes nerve to fight back: the significance of neural innervation of the bone marrow and spleen for immune function. Semin. Cell Dev. Biol..

[14] Bassi GS (2020). Anatomical and clinical implications of vagal modulation of the spleen. Neurosci. Biobehav. Rev..

[15] Lori A, Perrotta M, Lembo G, Carnevale D (2017). The spleen: a hub connecting nervous and immune systems in cardiovascular and metabolic diseases. Int. J. Mol. Sci..

[16] Noble BT, Brennan FH, Popovich PG (2018). The spleen as a neuroimmune interface after spinal cord injury. J. Neuroimmunol..

[17] Rosas-Ballina M (2008). Splenic nerve is required for cholinergic antiinflammatory pathway control of TNF in endotoxemia. Proc. Natl. Acad. Sci. USA.

[18] Felten DL, Ackerman KD, Wiegand SJ, Felten SY (1987). Noradrenergic sympathetic innervation of the spleen: I. Nerve fibers associate with lymphocytes and macrophages in specific compartments of the splenic white pulp. J. Neurosci. Res..

[19] Anagnostou VK (2007). Ontogeny of intrinsic innervation in the human thymus and spleen. J. Histochem. Cytochem..

[20] Murray K (2017). Neuroanatomy of the spleen: mapping the relationship between sympathetic neurons and lymphocytes. PLOS One.

[21] Bratton BO (2012). Neural regulation of inflammation: no neural connection from the vagus to splenic sympathetic neurons. Exp. Physiol..

[22] Ma B (2018). Distribution of non-myelinating Schwann cells and their associations with leukocytes in mouse spleen revealed by immunofluorescence staining. Eur. J. Histochem..

[23] Yuan A, Rao MV, Veeranna, Nixon RA (2012). Neurofilaments at a glance. J. Cell Sci..

[24] Wülfing C, Schuran FA, Urban J, Oehlmann J, Günther HS (2018). Neural architecture in lymphoid organs: Hard-wired antigen presenting cells and neurite networks in antigen entrance areas. Immun. Inflamm. Dis..

[25] Wülfing C, Günther HS (2015). Dendritic cells and macrophages neurally hard-wired in the lymph node. Sci. Rep..

[26] Beaulieu JM, Nguyen MD, Julien JP (1999). Late onset of motor neurons in mice overexpressing wild-type peripherin. J. Cell Biol..

[27] Pillai S, Cariappa A (2009). The follicular versus marginal zone B lymphocyte cell fate decision. Nat. Rev. Immunol..

[28] Kushwah R, Hu J (2011). Complexity of dendritic cell subsets and their function in the host immune system. Immunology.

[29] Godinho-Silva C, Cardoso F, Veiga-Fernandes H (2019). Neuro-immune cell units: a new paradigm in physiology. Annu. Rev. Immunol..

[30] Dustin ML (2012). Signaling at neuro/immune synapses. J. Clin. Invest..

[31] Hu D (2019). Immunofluorescence characterization of innervation and nerve-immune cell interactions in mouse lymph nodes. Eur. J. Histochem..

[32] Hua S (2016). Neuroimmune interaction in the regulation of peripheral opioid-mediated analgesia in inflammation. Front. Immunol..

[33] Ma B, von Wasielewski R, Lindenmaier W, Dittmar KE (2007). Immmunohistochemical study of the blood and lymphatic vasculature and the innervation of mouse gut and gut-associated lymphoid tissue. Anat. Histol. Embryol..

[34] Pacheco R, Prado CE, Barrientos MJ, Bernales S (2009). Role of dopamine in the physiology of T-cells and dendritic cells. J. Neuroimmunol..

[35] Kerage D, Sloan EK, Mattarollo SR, McCombe PA (2019). Interaction of neurotransmitters and neurochemicals with lymphocytes. J. Neuroimmunol..

[36] Razavi, R. *et al*. TRPV1^+^ sensory neurons control beta cell stress and islet inflammation in autoimmune diabetes. *Cell* **127**, 1123–1135 (2006). 10.1016/j.cell.2006.10.038 17174891

[37] Dubový P, Jančálek R, Kubek T (2013). Role of inflammation and cytokines in peripheral nerve regeneration. Int. Rev. Neurobiol..

[38] Fregnan F, Muratori L, Simões AR, Giacobini-Robecchi MG, Raimondo S (2012). Role of inflammatory cytokines in peripheral nerve injury. Neural Regen. Res..

[39] Chavan SS, Pavlov VA, Tracey KJ (2017). Mechanisms and therapeutic relevance of neuro-immune communication. Immunity.

[40] Voisin T, Bouvier A, Chiu IM (2017). Neuro-immune interactions in allergic diseases: novel targets for therapeutics. Int. Immunol..

[41] Pinho-Ribeiro FA (2018). Blocking neuronal signaling to immune cells treats streptococcal invasive infection. Cell.

[42] Johnson RL, Wilson CG (2018). A review of vagus nerve stimulation as a therapeutic intervention. J. Inflamm. Res..

[43] Ji H (2014). Central cholinergic activation of a vagus nerve-to-spleen circuit alleviates experimental colitis. Mucosal Immunol..

