# Supplementary material for: Distribution of nerve fibers and nerve-immune cell association in mouse spleen revealed by immunofluorescent staining
Source: Sci Rep. 2020 Jun 17;10:9850. doi: 10.1038/s41598-020-66619-0 (PMC7300136; doi:10.1038/s41598-020-66619-0)
Supplement: Supplementary file 1 — Supplementary Information. [file 41598_2020_66619_MOESM1_ESM.pdf]

Distribution of nerve fibers and nerve-immune cell association in mouse spleen revealed by immunofluorescent staining

Dailun Hu[1], Huda A.M. Al-Shalan[2,3], Zhongli Shi[1], Penghao Wang[2], Yongkang Wu[4], Philip K. Nicholls[2], Wayne K. Greene[2], Bin Ma[2*]

[1]Clinical College, Hebei Medical University, Shijiazhuang, 050020, China

[2] Medical, Molecular and Forensic Sciences, Murdoch University, Murdoch, 6149, Australia

[3] Department of Microbiology/Virology, College of Veterinary Medicine, Baghdad University, Baghdad, 10071, Iraq

[4]Department of Laboratory Medicine, West China Hospital, Sichuan University, Chengdu, 610041，China

Supplementary Materials

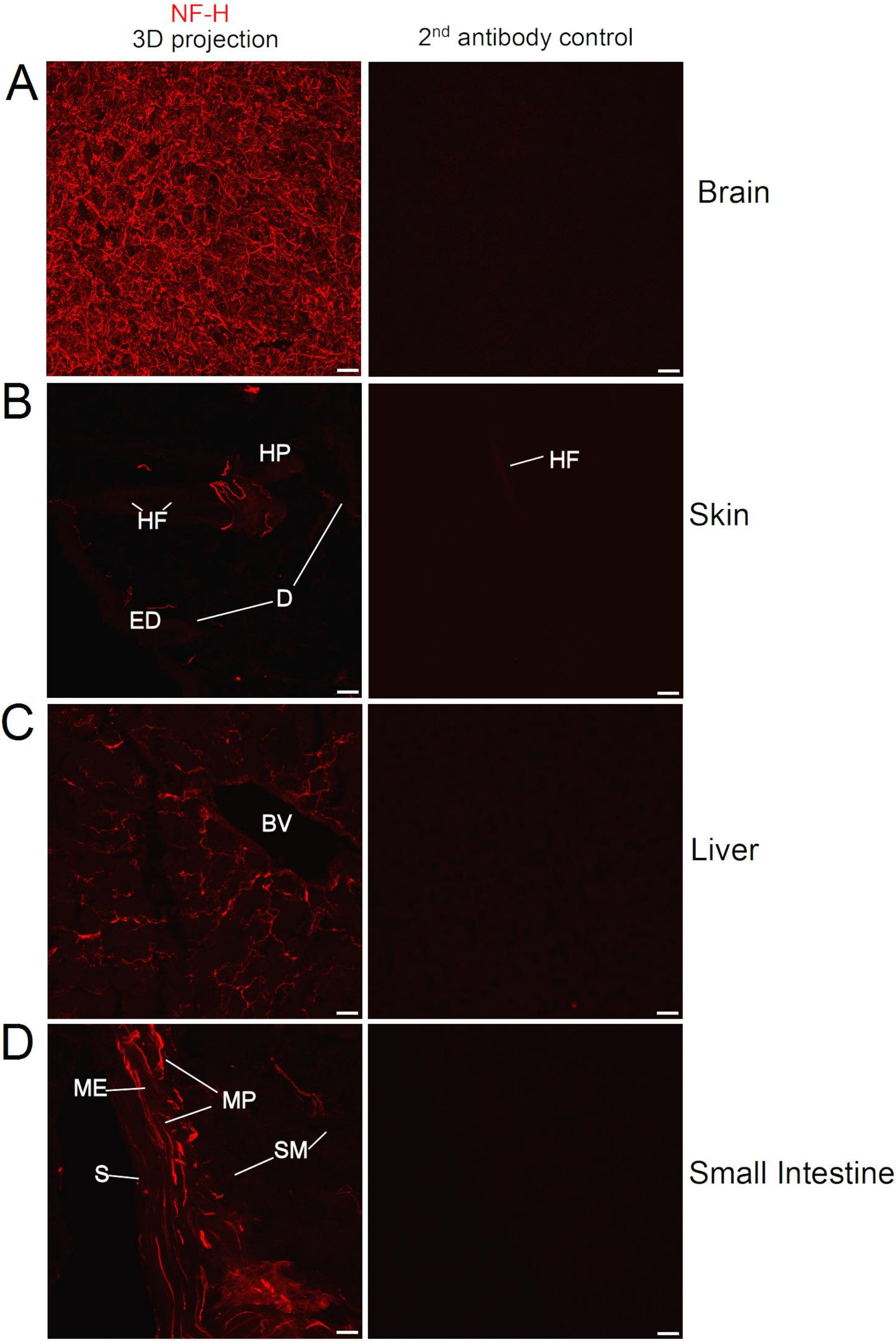
NF-H
3D projection
2nd antibody control
A
Brain
B
HP
HF
D
ED
HF
Skin
C
BV
Liver
D
ME
MP
SM
S
Small Intestine

Supplementary Fig. 1 Confocal images show NF-H staining in the sections from the brain (white matter), skin, liver, and small intestine of a C57BL/6 mouse. For 3D projection (left panels), maximal intensity projection of a Z-Stack (containing 13 optic slice) was performed. Stack size: 6.0 µm; optical slice interval: 0.50 µm. In the right panels, only secondary antibody, namely goat anti-rabbit Alexa 555, was applied. HF: hair follicle; HP: hair plexus; ED: epidermis; D: dermis; BV: blood vessel; S: serosa; MP: myenteric plexus; ME: muscularis externa; SM: submucosa; Objective lens: 40x; Scale bar: 20 µm

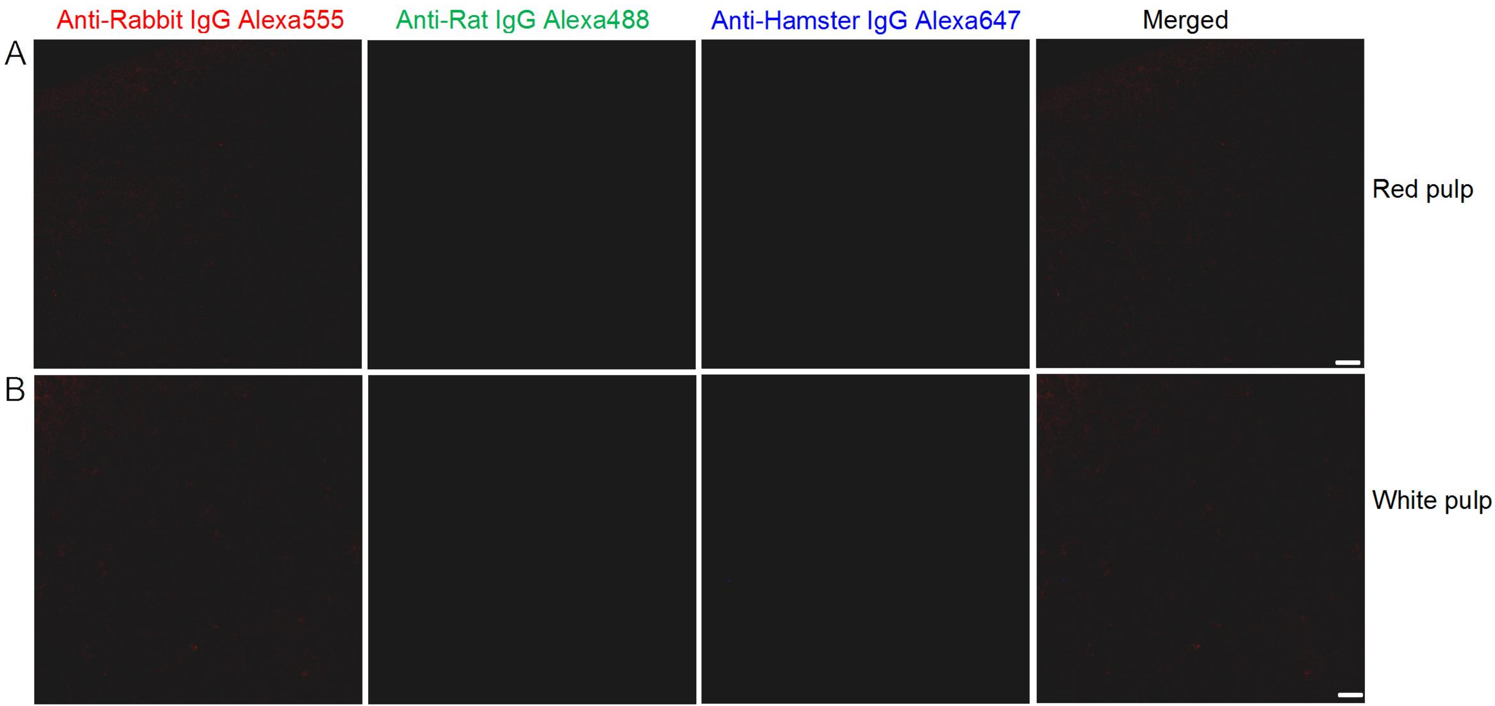
Anti-Rabbit IgG Alexa555
Anti-Rat IgG Alexa488
Anti-Hamster IgG Alexa647
Merged
A
B
Red pulp
White pulp

Supplementary Fig. 2 Confocal images of the splenic sections (from a C57BL/6 mouse) taken from the negative staining control experiments in which primary antibodies were omitted and only the three secondary antibodies were applied. The images were generated by using the same camera and device settings for other experiments. Objective lens: 40x. Scale bar: 20 µm

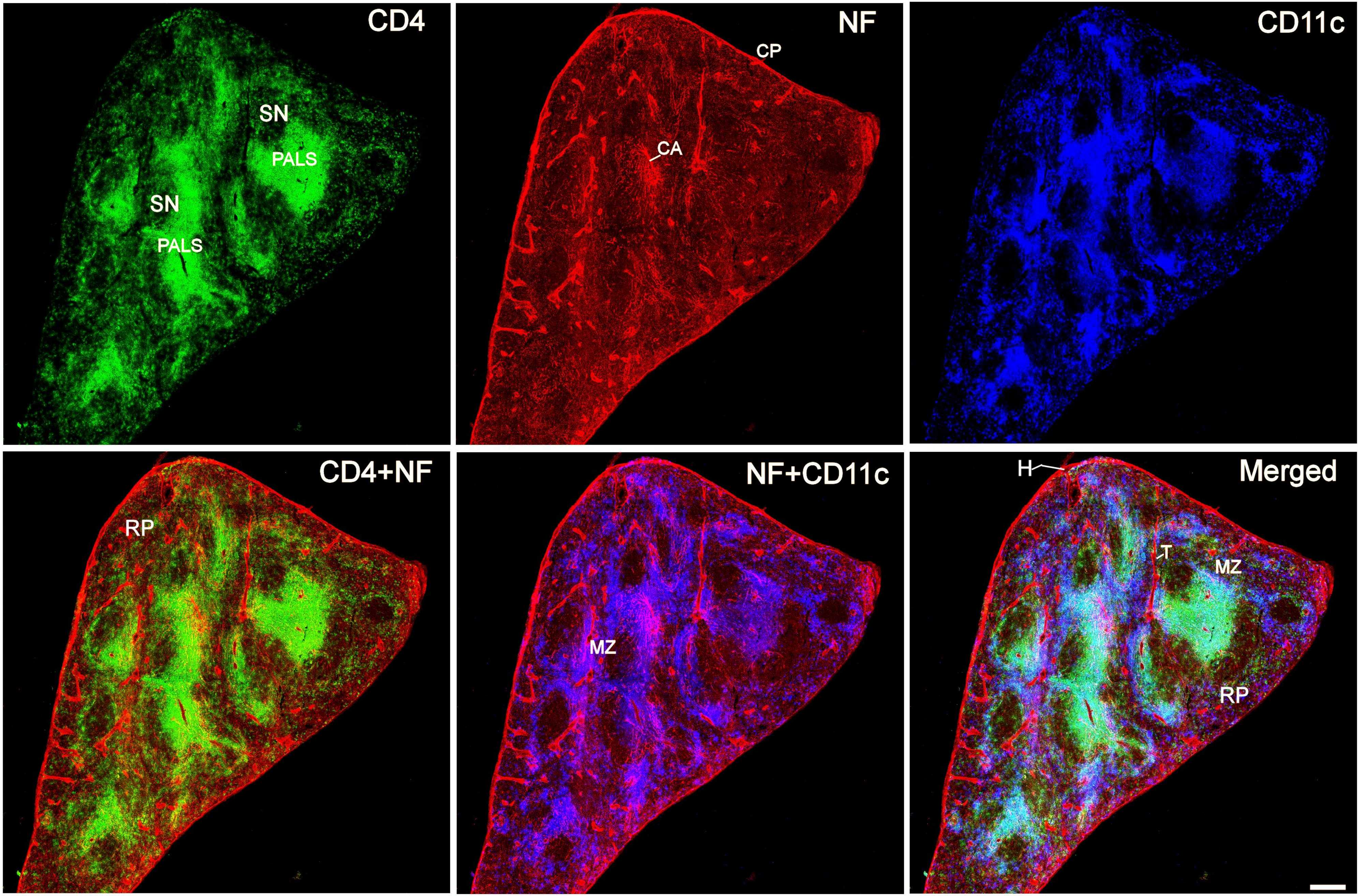
CD4
SN
PALS
SN
PALS
NF
CP
CA
CD11c
CD4+NF
RP
NF+CD11c
MZ
Merged
H
T
MZ
RP

Supplementary Fig. 3 Distribution of nerve fibers, T helper cells, and DCs in a C57BL/6 mouse spleen. Antibodies against CD4 (green), NF-H (red), and CD11c (blue) label mainly T helper cells, nerve fibers, and DCs, respectively. CA: central artery; SN: splenic nodule; PALS: periarteriolar lymphoid sheath; CP: capsule; RP: red pulp; MZ: marginal zone; T: trabecula; H: hilum; Objective lens: 40x; Scanning mode: Tile scan; Scale bar: 200 μm
